# Early versus late initiation of renal replacement therapy impacts mortality in patients with acute kidney injury post cardiac surgery: a meta-analysis

**DOI:** 10.1186/s13054-017-1707-0

**Published:** 2017-06-17

**Authors:** Honghong Zou, Qianwen Hong, Gaosi XU

**Affiliations:** 1 Medical Center of the Graduate School, Nanchang University, Nanchang, People’s Republic of China; 2 Science and Technology College, Jiangxi University of Traditional Chinese Medicine, Nanchang, People’s Republic of China; 3 Department of Nephrology, the Second Affiliated Hospital of Nanchang University, No. 1, Minde Road, Donghu District, Nanchang, 330006 People’s Republic of China

**Keywords:** Acute kidney injury, Cardiac surgery, Renal replacement therapy, Mortality, Early

## Abstract

**Background:**

Acute kidney injury (AKI) is a common clinical complication of cardiac surgery and increases mortality and hospitalization. We aimed to explore and perform an updated meta-analysis of qualitative and quantitative evaluations of the relationship between early renal replacement therapy (RRT) and mortality.

**Methods:**

We searched the Chinese Biomedical Database, the Cochrane Library, EMBASE, Global Health, MEDLINE and PubMed.

**Results:**

Fifteen studies (five randomized controlled trials (RCTs), one prospective cohort and nine retrospective cohorts) including 1479 patients were identified for detailed evaluation. The meta-analysis suggested that early RRT initiation reduced 28-day mortality (odds ratio (OR) 0.36; 95% confidence interval (CI) 0.23 to 0.57; *I* ^*2*^ 60%), and shortened intensive care unit (ICU) length of stay (LOS) (mean difference (MD) -2.50; 95% CI -3.53 to -1.47; *I* ^*2*^ 88%) and hospital LOS (MD -0.69; 95% CI -1.13 to -0.25; *I* ^*2*^ 88%), and also reduced the duration of RRT (MD -1.18; 95% CI -2.26 to -0.11; *I* ^*2*^ 69%), especially when RRT was initiated early within 12 hours (OR 0.23; 95% CI 0.08 to 0.63; *I* ^*2*^ 73%) and within 24 hours (OR 0.52; 95% CI 0.28 to 0.95; *I* ^*2*^ 58%) in patients with AKI after cardiac surgery.

**Conclusions:**

Early RRT initiation decreased 28-day mortality, especially when it was started within 24 hours after cardiac surgery in patients with AKI.

## Background

Acute kidney injury (AKI) as a complication following cardiac surgery is considered axiomatic and occurs in up to 45% of patients [1]. Although surgical, anesthetic and critical care advancements might efficiently decrease perioperative mortality and shorten intensive care unit (ICU) length of stay (LOS), it has not been confirmed in the reduction of the incidence of AKI, which increases the demand for postoperative renal replacement therapy (RRT) [2–4]. The initiation of RRT due to AKI has steadily increased over the past 15 years, which to some extent has enabled patients to survive longer [5–7], and decreased in-hospital mortality after surgeries [8–10]. Despite this, mortality due to AKI after cardiac surgery is an urgent problem to be solved, which presents challenges in clinical practice [11, 12].

Several meta-analyses relevant to this theme have been published in the past few years [13–16], which show the survival advantage of early initiation of RRT and recommend it for patients with AKI who undergo cardiac surgery, but there are some limitations in these studies; for instance, not all the referenced studies were based on cardiac surgery [14–16]. Furthermore, there were four further original studies [17–20] that were missed in the review by Liu et al. [13], which have been investigated in the present study.

To address these knowledge gaps, we conducted a new meta-analysis to update the present ones and provide the evidence to guide clinicians on this important issue. Furthermore, we categorized the definition of early and late RRT by time cutoffs, and examined some other clinical outcomes, e.g. the relationship between the modality of RRT and mortality. We specifically aimed to explore the impact of the time of starting early RRT on mortality in patients with AKI post cardiac surgery.

## Methods

### Search strategy

We searched the Chinese Biomedical Database, the Cochrane Library, EMBASE, Global Health, MEDLINE and PubMed for articles from November 1971 to August 2016. The predefined key search terms included “cardiac surgery” or “coronary artery surgery” or “coronary artery bypass grafting” or “cardiopulmonary bypass”, and “acute kidney injury” or “acute kidney failure” or “acute renal injury”, and “renal replacement therapy” or “hemodialysis”, and “early” or “late” or “time”. We reviewed the related research references at the same time.

### Study criteria

The inclusion criteria for studies were: (1) original research that related to early RRT initiation in adult patients with AKI after cardiac surgery, (2) articles that provided exact data on mortality in AKI and (3) articles that reported a clear comparison of early versus late RRT initiation with a direct effect on mortality. The exclusion criteria were: (1) duplication, (2) studies such as systemic reviews, meta-analyses, comments, case reports, animal experimental studies etc. and (3) studies of patients with pre-existing renal disease or who received RRT before undergoing cardiac surgery.

### Data extraction

Early and late RRT were defined on the basis of different criteria reported by the authors in their original research; we accepted a broad definition of early and late RRT and categorized this by time cutoffs (e.g., within a defined time after cardiac surgery, or development of urine output or a biochemical “start time” such as serum creatinine and blood urea levels etc.). The fifteen articles were divided into five groups according to early RRT initiation within 12 hours, within 24 hours, within 48 hours, within 72 hours or unclassified. Also, we summarized AKI diagnosis in the included articles and classified them on the basis of the 2012 *Kidney Disease: Improving Global Outcomes* (KDIGO) criteria. We intended to optimize the potential for identifying an effect associated with early or late RRT and to explore whether there is a relationship between the timing of starting early RRT and mortality after cardiac surgery with AKI. The modality of RRT was hemodialysis.

**Fig. 1:**
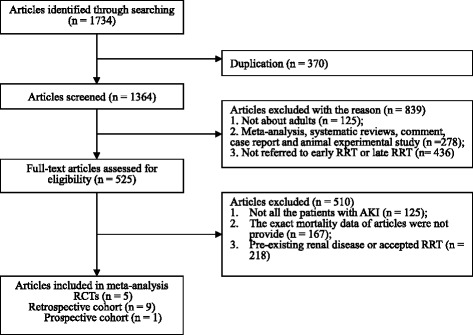
Study selection process. *RCT* randomized controlled trial, *RRT* renal replacement therapy, *AKI* acute kidney injury

Data were extracted independently by two investigators (HZ and QH). All potentially eligible citations that we searched were studied in detail to identify studies that satisfied the criteria. To identify duplicate records pertaining to a single study, we considered the PubMed database to take precedence; the details of the selection process are shown in Fig. 1. There would be a debate to reach consensus when there were disagreements. Data extraction included the first author’s name, year of publication, study design, RRT modality, definition of early and late RRT, number of patients, number of deaths due to AKI in post cardiac surgery, the mean and standard deviation or median of the ICU LOS, hospital LOS, duration of RRT and mechanical ventilation time: details are presented in Tables 1 and 2.

**Table 1:** The characteristics of early and late RRT studies included in the meta-analysis

Study	Year	Study design	RRT modality	Definition of early and late RRT		Mortality at 28 days Deaths/patients (*n*/*n* (%))		Total	OR(95% CI)	Quality score
				Early RRT	Late RRT	Early RRT	Late RRT			
Bouma [23]	2002	RCT	CRRT	UOP <30 mL/h within 12 h	Plasma urea level >40 mmol/L after 12 h	11/35 (31.4)	9/36 (25.0)	71	1.38 (0.49, 3.88)	M
Combes [17]	2015	RCT	CRRT/IHD	RRT initiation within 24 h post cardiac surgery in shock requiring high-dose catecholamine	Classic indication for RRT, life-threatening metabolic derangements	40/112 (35.7)	40/112 (35.7)	224	1.00 (0.58, 1.73)	H
Crescenzi [18]	2015	RCT	CRRT	Within 12 h of UOP <0.5 mL/kg/h	After 12 h on the basis of persistent (>6 h of UOP <0.5 mL/kg/h) oliguria	28/46 (60.9)	10/13 (76.9)	59	0.47 (0.11, 1.93)	L
Demirkilic [24]	2004	Retrospective cohort	CRRT	RRT initiation within 24 h after surgery when UOP <100 mL within consecutive 8 h	RRT initiation after 24 h post-surgery when Cr level exceeded 5 mg/dL or potassium level exceeded 5.5 mEq/L	8/34 (23.5)	15/27 (55.6)	61	0.25 (0.08, 0.74)	6
Durmaz [25]	2003	RCT	IHD	Serum Cr rise >10% from pre-op level within 48 h of surgery	Serum Cr rise >50% from pre-op level or UOP <400 mL over 24 h of surgery	1/21 (4.8)	7/23 (30.4)	44	0.11 (0.01, 1.03)	L
Elahi [26]	2004	Retrospective cohort	CRRT	RRT initiation within 24 h after surgery when UOP <100 mL within 8 h consecutively, despite furosemide infusion	RRT initiation after 24 h post-surgery when urea ≥30 mmol/L, serum Cr ≥250 mol/L or serum K ≥6 mEq/L	8/36 (22.2)	12/28 (42.9)	64	0.38 (0.13, 1.13)	6
Fernandez [27]	2011	Retrospective cohort	CRRT/IHD	Within 72 h after surgery	After 72 h post-surgery	54/101 (53.2)	82/102 (80.4)	203	0.28 (0.15, 0.52)	7
Helmut [28]	1987	Retrospective cohort	IHD	Before first hemodialysis within 48 h after surgery	Within 48 h after the first hemodialysis	9/21 (42.9)	10/15 (66.7)	36	0.38 (0.09, 1.49)	9
Iyem [29]	2009	Prospective cohort	CRRT	RRT initiation within 48 h when UOP ≤0.5 mL/kg/h post open-heart surgery	RRT initiation after 48 h when UOP ≤0.5 mL/ kg/h and 50% increase in baseline urea and Cr post open-heart surgery	5/95 (5.2)	6/90 (6.6)	185	0.78 (0.23, 2.64)	7
Ji [30]	2011	Retrospective cohort	CRRT	Within 12 h of UOP ≤0.5 mL/kg/h postoperatively	12 h after UOP ≤0.5 mL/kg/h postoperatively	3/34 (8.8)	9/24 (37.5)	58	0.16 (0.04, 0.68)	6
Kleinknecht [31]	1972	Retrospective cohort	IHD	Early and frequent hemodialysis to keep blood urea <200 mg/100 mL	Blood urea more than 350 mg/100 mL or severe presence of electrolyte abnormality	4/10 (40.0)	5/10 (50.0)	20	0.67 (0.11, 3.92)	7
Manche [32]	2008	Retrospective cohort	IHD	RRT initiation within 48 h after surgery when UOP <0.5 mL/kg/min	RRT initiation after 48 h post-surgery when all other supportive treatments failed	14/56 (25.0)	13/15 (87.0)	71	0.05 (0.01, 0.26)	6
Sugahara [33]	2004	RCT	CRRT	Within 12 h of UOP <30 mL/h	After 12 h of UOP <20 mL/h	2/14 (14.3)	12/14 (85.7)	28	0.03 (0.00, 0.23)	M
Szu-Yuan Li [19]	2014	Retrospective cohort	CRRT	Within 12 h of UOP <240 mL	UOP <240 mL after 12 h	44/97 (61.9)	33/45 (82.2)	142	0.30 (0.14, 0.65)	8
Xiao-Mei Yang [20]	2016	Retrospective cohort	IHD	RRT initiation within 24 h after surgery when AKI present in absence of traditional indications for RRT	RRT initiation after 24 h post-surgery when there were traditional indications for RRT	20/59 (33.9)	80/154 (51.9)	213	0.47 (0.25, 0.89)	7

*Abbreviations*: *RRT* renal replacement therapy, *Cr* creatinine, *UOP* urine output, *h* hours, *IHD* intermittent hemodialysis, *CRRT* continuous renal replacement therapy, *RCT* randomized controlled trial, *OR* odds ratio, *CI* confidence interval, *AKI* acute kidney injury, *pre-op* preoperative, *H* high quality: low risk of bias, *M*, medium quality: unclear risk of bias, *L*, low quality: high risk of bias

**Table 2:** Outcomes of early versus late RRT in patients with AKI after cardiac surgery

Study	ICU LOS (days)		Hospital LOS (days)		Duration of RRT (days)		Mechanical ventilation time (days)	
	Early	Late	Early	Late	Early	Late	Early	Late
Bouman [23]	13 ± 1.0	13.5 ± 1.5	27.0	35.5	3.9	2.9	1.0	12.0
Combes [17]	NR	NR	NR	NR	NR	NR	NR	NR
Crescenzi [18]	2.6 ± 5.5	2.2 ± 3.4	8.6 ± 7.7	8.2 ± 5.5	NR	NR	NR	NR
Demirkilic [24]	7.9 ± 1.3	12.4 ± 3.4	15.4 ± 4.0	20.9 ± 2.0	4.3 ± 1.5	4.6 ± 1.3	1.0 ± 0.6	3.0 ± 2.1
Durmaz [25]	1.6 ± 0.9	3.6 ± 2.9	8.9 ± 2.6	11.7 ± 4.8	NR	NR	NR	NR
Elahi [26]	8.5 ± 2.1	12.5 ± 5.3	15.4 ± 4.8	20.9 ± 7.3	4.6 ± 2.0	4.6 ± 11.4	NR	NR
Fernandez [27]	15.3 ± 15.4	27.9 ± 24.4	25.4 ± 28.6	38.2 ± 33.2	7.9 ± 10.7	12.5 ± 17.5	7.1 ± 9.8	10.7 ± 18.6
Helmut [28]	NR	NR	NR	NR	NR	NR	NR	NR
Iyem [29]	1.9 ± 1.0	3.7 ± 0.7	11.1 ± 4.6	17.1 ± 5.6	NR	NR	0.8 ± 0.6	0.8 ± 0.5
Ji [30]	5.0 ± 2.0	8.0 ± 2.0	13.0 ± 4.0	18.0 ± 6.0	2.4 ± 0.8	4.1 ± 1.1	NR	NR
Kleinknecht [31]	NR	NR	NR	NR	NR	NR	NR	NR
Manche [32]	NR	NR	NR	NR	NR	NR	NR	NR
Sugahara [33]	NR	NR	NR	NR	NR	NR	NR	NR
Szu-Yuan Li [19]	8.0	17.0	10.0	29.0	4.0	12.0	NR	NR
Xiao-Mei Yang [20]	12.5	14.0	38.0 ± 48.5	31.5 ± 33.0	6.6 ± 6.4	7.6 ± 7.4	7.3	8.5

Data are reported as mean ± standard deviation or median. *Abbreviations*: *RRT* renal replacement therapy, *AKI* acute kidney injury, *ICU* intensive care unit, *LOS* length of stay, *NR* not reported

### Quality assessment

**Fig. 2:**
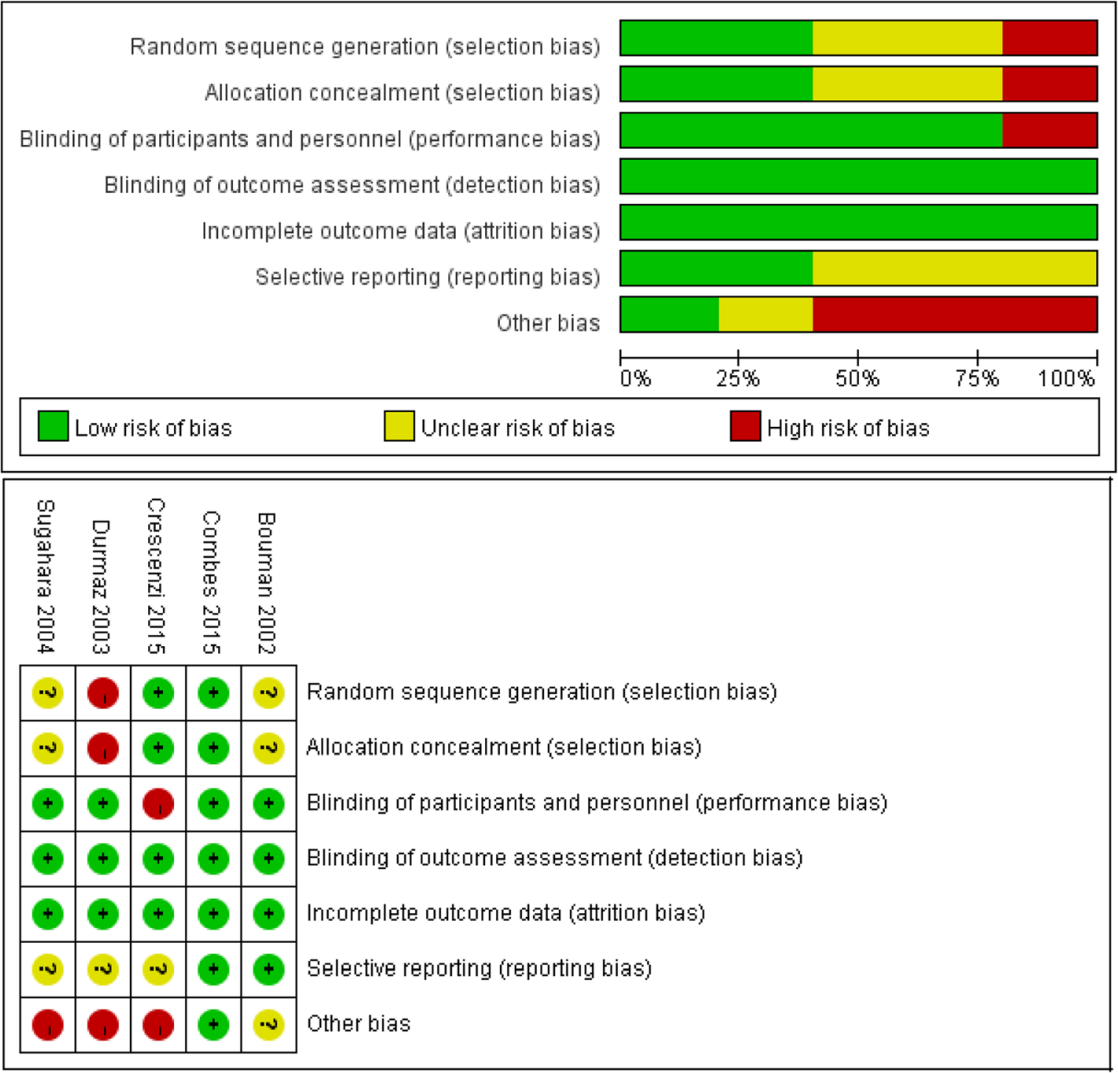
Risk of bias and summary of risk of bias

The assessment of study quality for the randomized controlled trials (RCTs) was performed using Review Manager (version 5.3) risk-of-bias tool, including four sections: selection, performance/detection, attrition and reporting bias (Fig. 2). The Newcastle-Ottawa Scale (NOS) (range 0 − 9 stars) was used to evaluate the cohort study quality (Table 1). For cohort studies, stars are awarded after evaluation of the three main categories of selection, comparability and outcomes: a study can be awarded no more than one star for each numbered option within the selection and exposure categories and no more than two stars can be awarded for comparability [21].

### Statistical analysis

The data were abstracted and analyzed using Review Manager (version 5.3) and STATA statistical software (version 12.0) to make the outcomes more comprehensive. Estimation of effect was performed using a random-effects model and was summarized by forest plot, with data expressed as odds ratio (OR) with 95% confidence interval (CI) for dichotomous outcomes and mean difference (MD) with 95% CI for continuous outcomes. A random-effects model was used to deal with data in light of the heterogeneity in the results and the clinical characteristics of the study, while a fixed-effects model was used when there was poor heterogeneity. Heterogeneity was evaluated by the *Q* statistic and *I* ^*2*^ tests, and low, moderate, and high heterogeneity was represented by thresholds of <25%, 25–75%, and > 75%, respectively [22]. *P* ≤ 0.05 was considered significant in all statistical tests. Subgroup and sensitivity analyses were used to explore the potential sources of heterogeneity. Egger and Begg test funnel plots were used to assess publication bias and the differences in the studies. Meta-regression was used to test the influence of baseline characteristics as potential effect modifiers, and the variables, including the year of publication, study design, RRT modality, definitions of early and late RRT, late RRT mortality rate ≥50% and classification of AKI.

## Results

### Description of included studies

Fifteen trials [17–20, 23–33] with a total of 1479 patients (771 patients receiving early RRT versus 708 patients receiving late RRT) ultimately met our criteria (Table 1); these included five RCTs [17, 18, 23, 25, 33], one prospective cohort study [29] and nine retrospective cohort studies [19, 20, 24, 26–28, 30–32]. The characteristics and methodological quality of all the included studies are shown in Table 1 and the outcomes in patients with AKI after cardiac surgery are shown in Table 2. Ten of the included articles were evaluated for study quality based on the assessment of the NOS [19, 20, 24, 26–32].

### Effect of early RRT on mortality

**Fig. 3:**
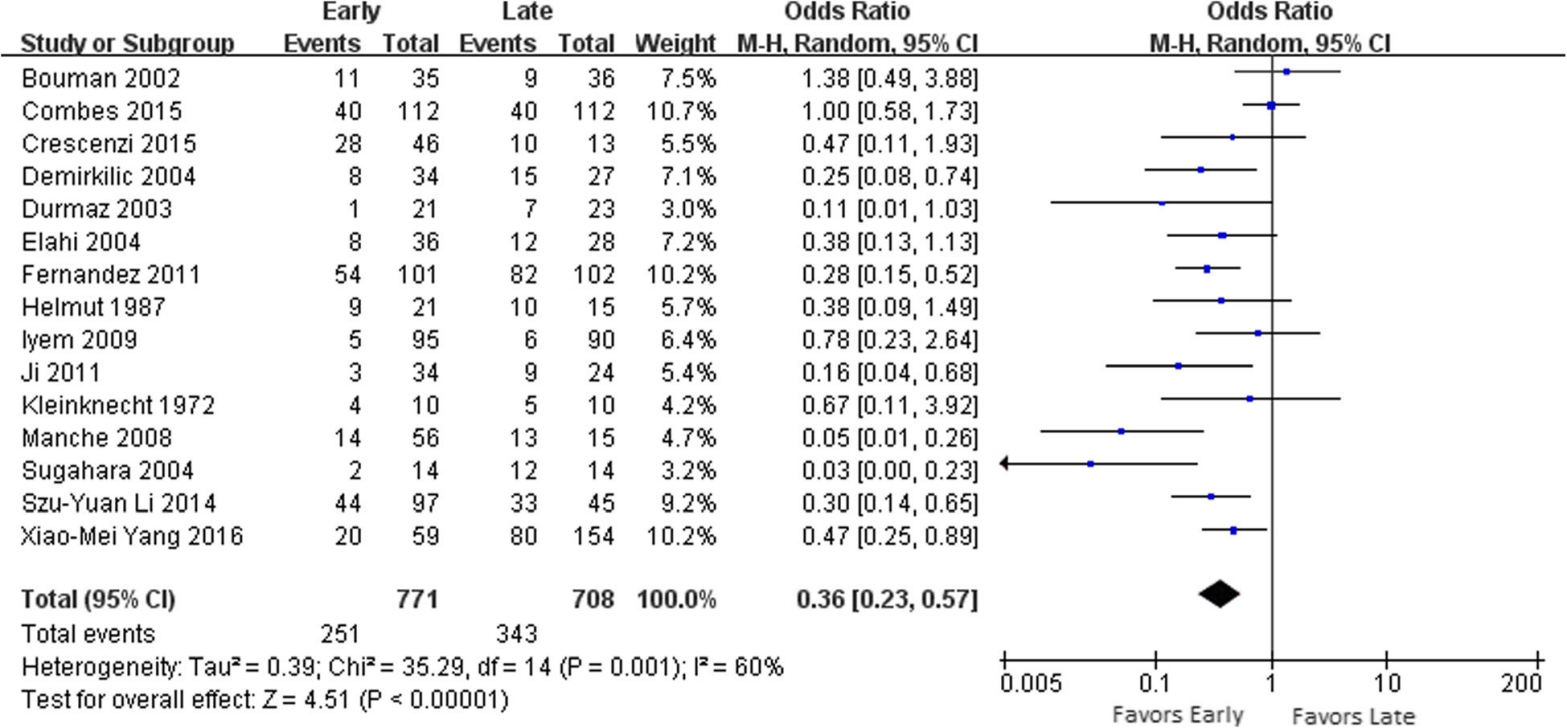
Forest plots of all 15 studies showed evidence of survival advantage of early renal replacement therapy initiation compared to late in analysis of mortality in patients with acute kidney injury after cardiac surgery

The effect of lower mortality was obvious in patients with AKI who underwent cardiac surgery and who received early RRT compared to patients who received late RRT (OR 0.36; 95% CI 0.23 to 0.57; *I* ^*2*^ 60%) (Fig. 3). Eight of the included articles showed that the ICU LOS (MD -2.50 days; 95% CI -3.53 to -1.47; *I* ^*2*^, 88%) (Table 3) and hospital LOS (MD -0.69 days; 95% CI -1.13 to -0.25; *I* ^*2*^ 88%) (Table 3) were also significantly decreased in early RRT, respectively, five included articles also showed that RRT duration was similarly decreased (MD -1.18 days; 95% CI -2.26 to -0.11; *I* ^*2*^ 69%) (Table 3). It was necessary to perform subgroup analysis to increase the reliability of the results and clarify the source of the high heterogeneity that we identified.

**Table 3:** Meta-analysis of outcomes of early versus late RRT in patients with AKI post cardiac surgery

Outcomes or subgroup analysis	Studies	Study reference number	Patients	OR﻿/MD (95% CI)	*I* ^*2*^	*P*
Primary outcomes: the effect of early versus late RRT on mortality						
Mortality at 28 days	15	[17–20, 23–33]	1479	0.36 (0.23, 0.57)]	60%	<0.01
Secondary outcomes: the relationship between early versus late RRT and mortality						
ICU LOS	8	[18, 23–27, 29, 30]	745	MD -2.50 (-3.53, -1.47)	88%	<0.01
Hospital LOS	8	[18, 20, 24–27, 29, 30]	887	MD -0.69 (-1.13, -0.25)]	88%	0.002
Duration of RRT	5	[20, 24, 26, 27, 30]	599	MD -1.18 (-2.26, -0.11)	69%	0.03
Subgroup analysis: the effect of the time of starting early RRT on mortality						
RRT initiation within 12 h	6	[18, 19, 23, 30, 32, 33]	429	0.23 (0.08, 0.63)	73%	0.005
RRT initiation within 24 h	4	[17, 20, 24, 26]	562	0.52 (0.28, 0.95)	58%	0.03
RRT initiation within 48 h	3	[25, 28, 29]	265	0.43 (0.17, 1.09)	15%	0.08
Subgroup analysis: the relationship between RRT modality and mortality						
CRRT	8	[18, 19, 23, 24, 26, 29, 30, 33]	668	0.36 (0.19, 0.67)	55%	0.001
IHD	5	[20, 25, 28, 31, 32]	384	0.27 (0.11, 0.66)	50%	0.004
CRRT/IHD	2	[17, 27]	427	0.53 (0.15, 1.86)	89%	0.32
Subgroup analysis: the relationship between study design and mortality						
RCTs	5	[17, 18, 23, 25, 33]	426	0.41 (0.14, 1.24)	74%	0.11
Cohort studies	10	[19, 20, 24, 26–32]	1053	0.33 (0.23, 0.46)	15%	<0.00001
Subgroup analysis: the relationship between AKI classification on the basis of 2012 KDIGO criteria and mortality						
KDIGO 1	7	[18, 19, 23, 25, 27, 29, 30]	762	0.40 (0.23, 0.69)	46%	0.001
KDIGO 2	2	[32, 33]	99	0.04 (0.01, 0.15)	0%	<0.00001
KDIGO 3	2	[24, 26]	125	0.31 (0.14, 0.66)	0%	0.003
Unclassified	4	[17, 20, 28, 31]	493	0.66 (0.41, 1.07)	23%	0.09

*Abbreviations*: *RRT* renal replacement therapy, *AKI* acute kidney injury, *RCT* randomized controlled trials, *OR* odds ratio, *CI* confidence interval, *ICU* intensive care unit, *CRRT* continuous renal replacement therapy, *IHD* Intermittent hemodialysis, *LOS* length of stay, *MD*, mean difference, *KDIGO* Kidney Disease: Improving Global Outcomes

### Subgroup analysis of early RRT and mortality

A subgroup analysis based on different time periods to initiate “early and late” RRT included thirteen articles [17–20, 23–26, 28–30, 32, 33] (Table 3), while one of the excluded article was not referred to time cutoffs [31], the other was the only one categorized to the group of 72 hours resulted in there was no comparability [27]. The outcomes of when to start early RRT were that in AKI after cardiac surgery, the incidence of mortality was significantly decreased by early initiation of RRT within 12 hours (OR 0.23; 95% CI 0.08 to 0.63; *I* ^*2*^ 73%) and within 24 hours (OR 0.52; 95% CI 0.28 to 0.95; *I* ^*2*^ 58%) compared to initiation within 48 hours (OR 0.43; 95% CI 0.17 to 1.09; *I* ^*2*^ 15%). In other words, early RRT initiation within 24 hours significantly reduced mortality compared to initiation after 24 hours in patients with AKI post cardiac surgery.

We also analyzed a subgroup based on RRT modality (Table 3): the impact of RRT modality on mortality was that the modality, whether continuous RRT (CRRT) (OR 0.36; 95% CI 0.19 to 0.67; *I* ^*2*^ 55%) or intermittent hemodialysis (IHD) (OR 0.27; 95% CI 0.11 to 0.66; *I* ^*2*^ 50%), had no effect on mortality in patients with AKI post cardiac surgery.

Furthermore, in another subgroup analysis based on study design (Table 3), in cohort studies there was a statistically significant decrease in mortality among patients who received early RRT (OR 0.33; 95% CI 0.23 to 0.46; *I* ^*2*^ 15%), while the decrease in mortality in the RCTs (OR 0.41; 95% CI 0.14 to 1.24; *I* ^*2*^ 74%) was not statistically significant.

Finally, there was insignificant statistics in the subgroup of AKI classification (Table 3), the outcomes of different classifications on AKI dignosis impacted on the mortality as following KDIGO 1 (OR 0.40; 95% CI 0.23 to 0.69; *I* ^*2*^ 46%), KDIGO 2 (OR 0.04; 95% CI 0.01 to 0.15; *I* ^*2*^ 0%), KDIGO 3 (OR 0.31; 95% CI 0.14 to 0.66; *I* ^*2*^ 0%) and unclassified (OR 0.66; 95% CI 0.41 to 1.07; *I* ^*2*^ 23%).

### Sensitivity analysis and publication bias

**Fig. 4:**
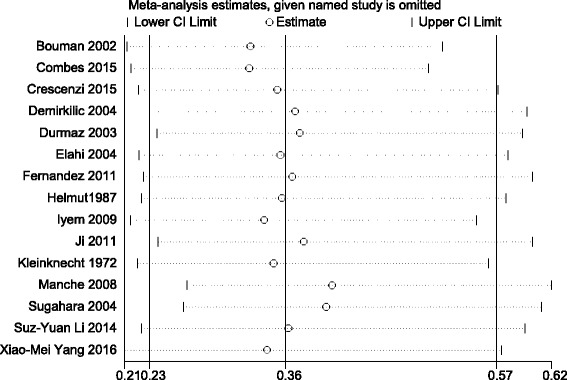
Sensitivity analysis shows the meta-analysis has low sensitivity and satisfactory stability for analysis of patients with acute kidney injury after cardiac surgery

**Fig. 5:**
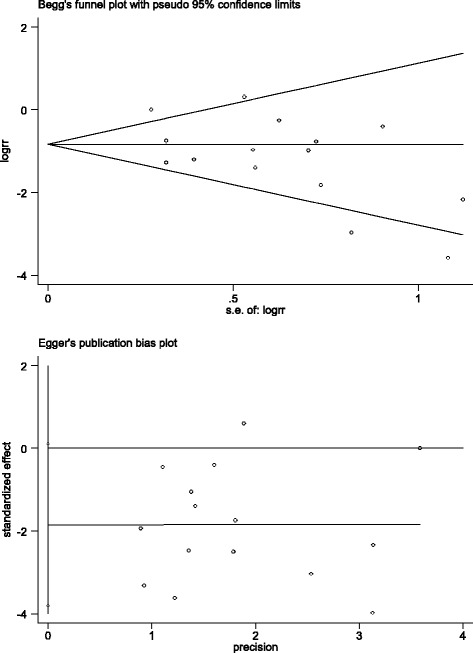
Publication bias according to Egger and Begg test funnel plots

Sensitivity analysis indicated that the meta-analysis has low sensitivity and high stability in analysis of patients with AKI who had undergone cardiac surgery, which is demonstrated in Fig. 4. The Egger and Begg test funnel plots were used to explore publication bias (Fig. 5), the Egger linear regression test (*P* > 0.062) and the Begg rank correlation test (*Pr* > |z| = 0.113), which provided no evidence of substantial publication bias in this meta-analysis.

### Sources of heterogeneity and meta-regression

We performed meta-regression to investigate the sources of heterogeneity. The association between early RRT initiation and mortality was not influenced by year of publication (*P* = 0.949), either in study design (*P* = 0.114), RRT modality (*P* = 0.366) or a late RRT mortality rate ≥50% (*P* = 0.087). Stratification of studies according to the definition of AKI showed no impact on mortality when early and late RRT criteria were defined on the basis of time cutoffs and categorized by urine output, creatinine and blood urea (*P* = 0.541).

## Discussion

In the present meta-analysis of 15 articles involving 1479 adult patients with AKI who underwent cardiac surgery, we found that postoperative administration of early RRT decreased mortality and reduced the ICU LOS, hospital LOS and duration of RRT. Postoperative AKI is a common complication; the risk of death associated with AKI is proportional to its severity, with the highest rate in patients requiring hemodialysis post cardiac surgery [34–36], therefore, it is urgent that we distinguish AKI from the multitudinous complications and deal with it. In our current analysis, positive renal protective actions like initiating early RRT efficiently decreased the mortality due to AKI after cardiac surgery. The advantages for early RRT might be in decreasing the occurrence of life-threatening complications such as uremia, acidosis, volume overload and hyperkalemia. Interventions that attain the balance of solute clearance and fluid before progression to more serious disease also effectively attenuate kidney-specific and non-kidney organ injury when compared to late RRT.

However, there were also passive or controversial studies pertaining to the effect of early RRT, such as some studies that suggested that it was not necessary for clinicians to perform early RRT. Some were opposed to early RRT because it could expose patients to potential harms such as hemorrhage, bacteremia, thrombosis, intradialytic hypotension, clearance of trace elements and hypersensitivity to the extracorporeal circuit or antibiotics, which could lead to added resource utilization [37]. Our analysis produced the primary outcome that the initiation of early RRT showed evidence of survival advantages compared to late RRT. What is more, in our second outcome we found that early RRT initiation shortened the ICU LOS, hospital LOS and duration of RRT. Furthermore, in the subgroup analysis, early RRT initiation within 24 hours was associated with low mortality when compared to RRT initiation after 24 hours in patients with AKI post cardiac surgery.

To explore the sources of the heterogeneity, first, we performed the subgroup analysis based on RRT modality and study design, respectively, which were not statistically significant. Second, we completed sensitivity analysis, which indicated that our meta-analysis had low sensitivity and satisfactory stability. Egger and Begg test funnel plots showed no publication bias in this meta-analysis: however, despite this we should not jump rashly to the conclusion of no publication bias, because the *P* value for the Egger linear regression test (*P* > 0.062) was close to 0.05. Last but not least, meta-regression included five variables that were not heterogenous. As for the high heterogeneity of ICU LOS, hospital LOS and duration of RRT, not all the articles provided original data for the mean and standard deviation, which might affect the heterogeneity.

There were several potential limitations in the meta-analysis. First, the definition of early RRT criteria was different in the included studies, which may have led to the difference in the requirement for RRT and the subsequent therapeutic results in patients with cardiac-surgery-related AKI [38]. Second, the sample size in each of the included five RCTs is relatively small, so it is necessary to perform large, multi-centered RCTs to support the present results. Although the AKIKI trial [39] and ELAIN trial [40] were performed in critically ill patients, the standard definition of AKI and indication for RRT may be applied to patients with AKI post cardiac surgery. Third, there are limitations to retrospective trials such as the sample size, and therefore they may not be representative and the risk of recall bias may be higher.

## Conclusions

Cardiac-surgery-associated AKI probably benefits from early RRT initiation, which would decrease 28-day mortality and shorten the ICU LOS, hospital LOS and duration of RRT. What is more, early RRT initiation within 24 hours showed evidence of a survival advantage when compared to initiation after 24 hours in patients with AKI post cardiac surgery.

## Abbreviations

AKI: Acute kidney injury
CI: Confidence interval
Cr: Creatinine
CRRT: Continuous renal replacement therapy
h: Hours
ICU: Intensive care unit
IHD: Intermittent hemodialysis
KDIGO: Kidney Disease: Improving Global Outcomes
LOS: Length of stay
MD: Mean difference
NOS: Newcastle-Ottawa Scale
NR: Not reported
OR: Odds ratio
RCTs: Randomized controlled trials
RRT: Renal replacement therapy
UOP: Urine output
