# Correction to: Early versus late initiation of renal replacement therapy impacts mortality in patients with acute kidney injury post cardiac surgery: a meta-analysis

**DOI:** 10.1186/s13054-018-2065-2

**Published:** 2019-04-25

**Authors:** Honghong Zou, Qianwen Hong, Gaosi XU

**Affiliations:** 1 Department of Nephrology, the Second Affiliated Hospital of Nanchang University, No. 1, Minde Road, Donghu District, Nanchang, 330006 People’s Republic of China; 2 Science and Technology College, Jiangxi University of Traditional Chinese Medicine, Nanchang, People’s Republic of China

## Correction

After publication of this article [1], it was noticed that the affiliations of Honghong Zou were incorrect. The sole affiliation should be, “Department of Nephrology, the Second Affiliated Hospital of Nanchang University, People’s Republic of China” and can be seen in the author details of this correction.
